# Effect of Atomoxetine on Hyperactivity in an Animal Model of Attention-Deficit/Hyperactivity Disorder (ADHD)

**DOI:** 10.1371/journal.pone.0108918

**Published:** 2014-10-01

**Authors:** Su Jin Moon, Chang Ju Kim, Yeon Jung Lee, Minha Hong, Juhee Han, Geon Ho Bahn

**Affiliations:** 1 Department of Psychiatry, Daedong Hospital, Daegu, Korea; 2 Department of Physiology, Kyung Hee University School of Medicine, Seoul, Korea; 3 Department of Psychiatry, Kyung Hee University School of Medicine, Seoul, Korea; 4 Department of Psychiatry, Dankook University Medical College, Cheonan, Korea; Alexander Fleming Biomedical Sciences Research Center, Greece

## Abstract

**Background:**

Hyperactivity related behaviors as well as inattention and impulsivity are regarded as the nuclear symptoms of attention-deficit/hyperactivity disorder (ADHD).

**Purpose:**

To investigate the therapeutic effects of atomoxetine on the motor activity in relation to the expression of the dopamine (DA) D2 receptor based on the hypothesis that DA system hypofunction causes ADHD symptoms, which would correlate with extensive D2 receptor overproduction and a lack of DA synthesis in specific brain regions: prefrontal cortex (PFC), striatum, and hypothalamus.

**Methods:**

Young male spontaneously hypertensive rats (SHR), animal models of ADHD, were randomly divided into four groups according to the daily dosage of atomoxetine and treated for 21 consecutive days. The animals were assessed using an open-field test, and the DA D2 receptor expression was examined.

**Results:**

The motor activity improved continuously in the group treated with atomoxetine at a dose of 1 mg/Kg/day than in the groups treated with atomoxetine at a dose of 0.25 mg/Kg/day or 0.5 mg/Kg/day. With respect to DA D_2_ receptor immunohistochemistry, we observed significantly increased DA D_2_ receptor expression in the PFC, striatum, and hypothalamus of the SHRs as compared to the WKY rats. Treatment with atomoxetine significantly decreased DA D_2_ expression in the PFC, striatum, and hypothalamus of the SHRs, in a dose-dependent manner.

**Conclusion:**

Hyperactivity in young SHRs can be improved by treatment with atomoxetine via the DA D2 pathway.

## Introduction

Attention-deficit/hyperactivity disorder (ADHD) is a common childhood and adolescent disorder that affects 5–10% of school-aged children worldwide [1] and can affect 3–5% of the adult population [2]. In spite of the high prevalence of ADHD, its exact pathophysiology has not yet been established. Various biologic factors have been suggested to contribute to the etiology of the disease. The “dopaminergic hypothesis” is the most widely accepted hypothesis by researchers for understanding the ADHD pathophysiology [3]. It is based on dysregulation in dopaminergic neurotransmission causing behavioral alterations in both ADHD and in the spontaneously hypertensive rat (SHR), which has been used as an ADHD animal model [4]. Initial functional MRI studies showed decreased activation of the dopamine (DA) pathway [5] and the DA hypothesis suggested that DA deficits in specific brain regions, such as cortical areas and/or the striatum, could produce ADHD symptoms [6]. DA receptors are the main determinants of the dopamine pathway and are divided into 2 classes [7]. D1-like receptor subtypes, D1 and D5, couple to the G protein Gs and activate adenylyl cyclase. The other receptor subtypes belong to the D2-like subfamily (D2, D3, and D4) and are prototypic of G-protein coupled receptors that inhibit adenylyl cyclase. In particular, the DA D2 receptor is closely associated with neuropsychiatric disorders, including ADHD [8], schizophrenia [9], and depression [10]. The DA D2 receptor belongs to the G protein-coupled receptor family and its activation inhibits synaptogenesis [11]. Enhancement of DA D_2_ receptor function, induced by increased expression of the Gi-α protein, accounts for the decreased release of DA in the striatum of SHRs [7]. Further, Bowton et al. [8] demonstrated that dysregulation of dopamine transporters, via DA D2-autoreceptors, triggers an anomalous dopamine efflux associated with ADHD.

It has been known that the cerebral stimulants are closely related to the DA pathway; however, atomoxetine has been identified as a selective inhibitor of norepinephrine transporter that increases the extracellular concentrations of norepinephrine and also dopamine in the prefrontal cortex (PFC) [12]. Although ATX increased DA concentrations in the PFC 3-fold, the selectivity of ATX for NE effects in the PFC was also demonstrated [12]. Therefore, it has been recognized that ATX may have effects on attention, anxiety, and social affect in addition to hyperactivity [13]. Recent studies reported that atomoxetine produced changes in impulsivity via D2/D3 receptors [14].

There are several reports about the effects of atomoxetine on inattention and impulsivity; however, studies on the effect of atomoxetine on hyperactivity, especially via D2 receptors, are very rare [15], [16]. The pathological mechanisms of ADHD with respect to DA synthesis, DA receptors, and the effects of atomoxetine on DA synthesis and DA receptors still remain unclear.

Therefore, we investigated the therapeutic effects of atomoxetine on the motor activity in relation to the expression of the DA D_2_ receptor in the SHR animal model of ADHD based on the hypothesis that DA system hypofunction causes ADHD symptoms, which would correlate with extensive D_2_ receptor overproduction and a lack of DA synthesis in specific brain regions such as PFC, striatum, and hypothalamus.

## Materials and Methods

### Experimental animals and treatments

The SHR is a valid and currently accepted model for the study of ADHD [17]. The SHRs are known to display hyperactivity, impulsivity, poor sustained attention, and deficits in learning and memory processes in comparison with normotensive Wistar-Kyoto (WKY) rats [4]. Male animals, weighing 180±5 g (6-week-old), were obtained from a commercial breeder (Orient Co., Seoul, Korea): SHRs served as ADHD rats, and WKY rats as controls. Each animal was housed under controlled temperature (23±2°C) and lighting (08∶00–20∶00) conditions, with food and water made available *ad libitum*. All experimental procedures were performed in accordance with the animal care guidelines of the National Institutes of Health (NIH) and the Korean Academy of Medical Sciences. The protocol was approved by the Ethics Committee of Kyung Hee University and the? Institutional Animal Care and Use Committee (Permit Number: 13-038).

The SHRs were randomly divided into four groups (n = 10 in each group): the untreated SHR group; the 0.25 mg/kg/day atomoxetine-treated SHR group; the 0.5 mg/kg/day atomoxetine-treated SHR group; and the 1 mg/kg/day atomoxetine-treated SHR group. The control group comprised of 10 WKY rats.

The rats in the atomoxetine-treated groups received atomoxetine (Strattera, Eli Lilly Co., Indianapolis, IN, USA) orally once a day for 21 consecutive days, at the respective dosage. The rats in the control group and the untreated SHR group received an equal amount of distilled water for the same duration.

### Open-field test

Open-field test has often been used for the assessment of behaviors such as motor activity [18]. Ambulatory and rearing activities in the open field environment were significantly higher in juvenile, stroke-prone SHRs than in WKY rats [19]. The open-field test procedure was performed according to a previous report [20], testing all of the groups four times: one day before the start of the atomoxetine (or water) treatment, one week after the start of treatment, two weeks after the start of treatment, and three weeks after the start of treatment. The open field box was composed of a 100 cm×100 cm×40 cm wooden enclosure, and the field was divided into 25 squares (20×20 cm), defined as 9 central and 16 peripheral squares. Each rat was placed in the central square and allowed to move freely and explore the environment for 1 minute. Next, the number of squares that the rat crossed was recorded for 5 minutes. The entire area was cleaned between tests.

### Tissue preparation

All of the rats were sacrificed at 21 days after the initiation of drug treatment. The animals were anesthetized with Zoletil 50 (10 mg/kg, i.p.; Vibac Laboratories, Carros, France), transcardially perfused with 50 mM phosphate-buffered saline (PBS), and fixed with a freshly-prepared solution of 4% paraformaldehyde in 100 mM phosphate buffer (PB, pH 7.4). Next, the brains were dissected and postfixed in the same fixative overnight, and transferred into a 30% sucrose solution for cryoprotection. Coronal sections of 40 µm thickness were cut with a freezing microtome (Leica, Nussloch, Germany). Ten slice sections were obtained from each rat, with each slice containing the prefrontal cortex (PFC), striatum, and hypothalamus.

### DA D2 receptor immunohistochemistry

Immunohistochemistry was performed to immunolabel the DA D_2_ receptors in the PFC, striatum, and hypothalamus. Free-floating tissue sections were incubated overnight with mouse anti-DA D_2_ receptor antibody (1∶500, Santa Cruz Biotechnology, Santa Cruz, CA, USA) and then incubated for 1 hour with biotinylated anti-mouse secondary antibody (1∶200, Vector Laboratories, Burlingame, CA, USA). The sections were subsequently incubated with avidin-biotin-peroxidase complex (Vector Laboratories) for 1 hour at room temperature. To visualize immunoreactivity, the sections were incubated in a solution of 0.05% 3, 3-DAB and 0.01% H_2_O_2_ in 50 mM Tris-buffer (pH 7.6) for approximately 3 minutes. The sections were then washed three times with PBS and mounted onto gelatin-coated slides. The slides were air-dried overnight at room temperature, and cover slips were mounted using Permount.

We calculated the optical density of DA D_2_ receptor-immunoreactive fibers in the PFC, striatum, and hypothalamus in each slice. First, the areas of PFC, striatum, and hypothalamus in each slice were measured using the Image-Pro Plus computer-assisted image analysis system (Media Cybernetics Inc., Silver Spring, MD, USA) attached with a light microscope (Olympus, Tokyo, Japan). Next, the DA D_2_ receptor-immunoreactive fiber densities were measured in 100×100 µm square images, of the PFC, striatum, and hypothalamus, using an image analyzer (Multiscan, Fullerton, CA, USA). To estimate the DA D_2_ receptor-staining densities, the optical densities were corrected for the nonspecific background density, which was measured in completely denervated areas of the striatum.

### Data analysis

Statistical analysis was performed using one-way ANOVA followed by Duncan's post-hoc test, and the results were expressed as the mean ± standard error of the mean (SEM). The limit for statistical significance was set at *p*<0.05.

## Results

**Figure 1:**
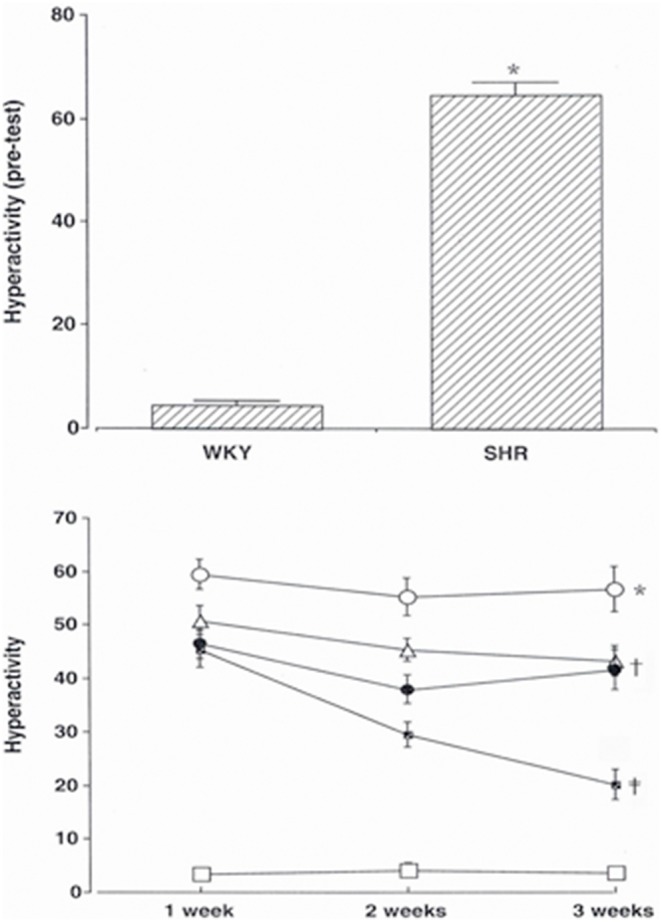
Effect of atomoxetine on hyperactivity in the open field test. Upper: Pre-open field test. Lower: Open field test for experimental periods. (□) Control group, (○) SHR group, (Δ) SHR and 0.25 mg/kg atomoxetine-treatd group, (•) SHR and 0.5 mg/kg atomoxetine-treatd group, (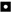) SHR and 1 mg/kg atomoxetine-treatd group. * represents p<.05 compared to the control group. ^†^ represents p<.05 compared to the SHR group. ^‡^ represents p<.05 compared to the SHR and 0.25 mg/kg atomoxetine-treated group.

The open-field tests were carried out one day before the start of atomoxetine and water treatment, one week after the start of atomoxetine and water treatment, two weeks after the start of atomoxetine and water treatment, and three weeks after the start of atomoxetine and water treatment. At one day before the start of treatment, the activity score in controls was 4.60±0.79, and the activity score in SHRs was 64.52±2.37 (Fig. 1). The activity scores at one week after the start of treatment, two weeks after the start of treatment, and three weeks after the start of treatment were 3.60±0.84, 4.30±1.22, and 3.70±0.91, respectively in the control rats; 59.50±2.82, 55.30±3.56, and 56.70±4.18, respectively in the untreated SHR group; 50.90±2.82, 45.50±2.11, and 43.30±2.88, respectively in the 0.25 mg/kg/day atomoxetine-treated SHR group; 46.70±2.81, 38.00±2.69, and 41.70±3.74, respectively in the 0.5 mg/kg/day atomoxetine-treated SHR group; and 45.50±3.47, 29.50±2.28, and 20.10±2.81, respectively in the 1 mg/kg/day atomoxetine-treated SHR group. These results demonstrated that the activity of the SHRs was higher than that of the control rats. Atomoxetine treatment decreased the activity of SHRs in a time- and dose-dependent manner (*F_(4,45)_* = 42.178, *p*<0.001).

**Figure 2:**
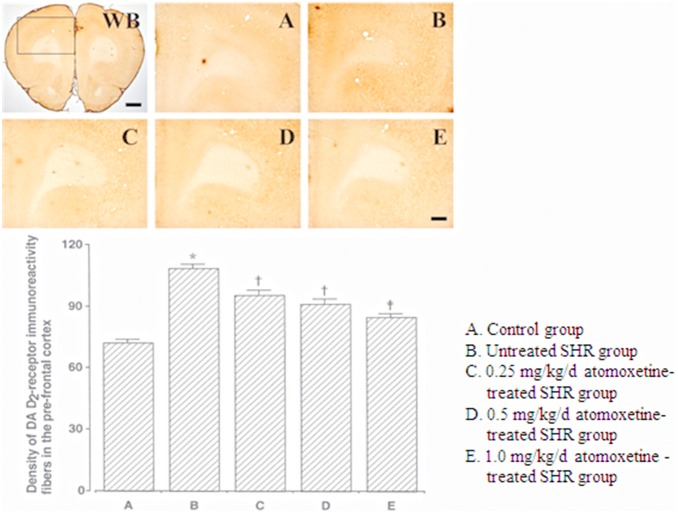
Effect of atomoxetine on DA D_2_ receptor expression in the pre-frontal cortex. Upper: Photomicrographs of DA D_2_ receptor expression in the pre-frontal cortex. WB: Whole brain. The sections were stained for DA D_2_ receptor immunoreactivity (brown). The scale bars represent 50 µm (WB) and 200 µm (A–E). Lower: DA D_2_ receptor expression in each group. *p<0.05 compared to the control group. ^†^ *p*<0.05 compared to the untreated ADHD group. ^‡^ *p*<0.05 compared to the untreated ADHD and 0.25 mg/kg/d atomoxetine-treated ADHD groups. The data have been presented as mean ± SEM.

The DA D_2_ receptor-immunoreactive fiber density in the PFC was 72.20±1.77 in the control group, 108.50±1.72 in the untreated SHR group, 95.00±2.34 in the 0.25 mg/kg/day atomoxetine-treated SHR group, 90.60±2.55 in the 0.5 mg/kg/day atomoxetine-treated SHR group, and 84.40±1.66 in the 1 mg/kg/day atomoxetine-treated SHR group (Fig. 2). These results indicated that the DA D_2_ receptor-immunoreactive fiber density in the PFCs of SHRs was significantly higher than that in the PFCs of controls, and atomoxetine treatment significantly and dose-dependently decreased the DA D_2_ receptor-immunoreactive fiber density in the PFC (*F_(4,40)_* = 44.921, *p* = 0.001).

**Figure 3:**
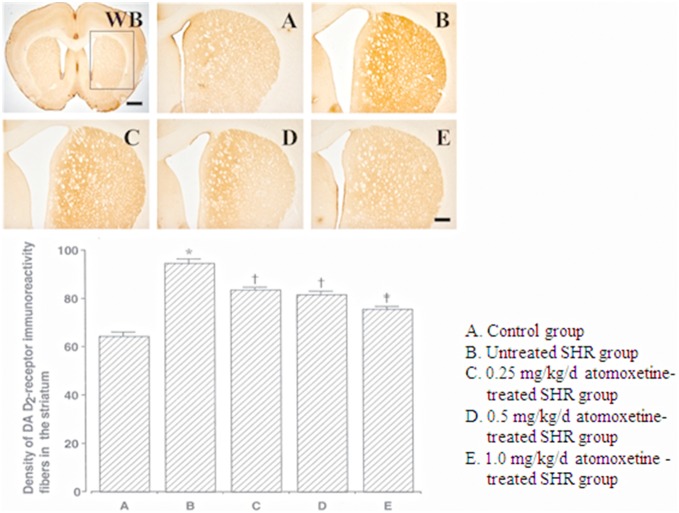
Effect of atomoxetine on DA D_2_ receptor expression in the striatum. Upper: Photomicrographs of DA D_2_ receptor expression in the striatum. WB: Whole brain. The sections were stained for DA D_2_ receptor immunoreactivity (brown). The scale bars represent 50 µm (WB) and 200 µm (A–E). Lower: DA D_2_ receptor expression in each group. *p<0.05 compared to the control group. ^†^ *p*<0.05 compared to the untreated ADHD group. ^‡^ *p*<0.05 compared to the untreated ADHD and 0.25 mg/kg/d atomoxetine-treated ADHD groups. The data have been presented as mean ± SEM.

The DA D_2_ receptor-immunoreactive fiber density in the striatum was 64.40±1.57 in the control group, 94.50±1.92 in the untreated SHR group, 83.60±0.92 in the 0.25 mg/kg/day atomoxetine-treated SHR group, 81.80±1.10 in the 0.5 mg/kg/day atomoxetine-treated SHR group, and 75.70±0.94 in the 1 mg/kg/day atomoxetine-treated SHR group (Fig. 3). These results showed that the DA D_2_ receptor-immunoreactive fiber density in the striatum of SHRs was significantly higher than that in the striatum of the controls, and atomoxetine treatment significantly and dose-dependently decreased the DA D_2_ receptor-immunoreactive fiber density in the striatum (*F_(4,40)_* = 47.480, *p*<0.001).

**Figure 4:**
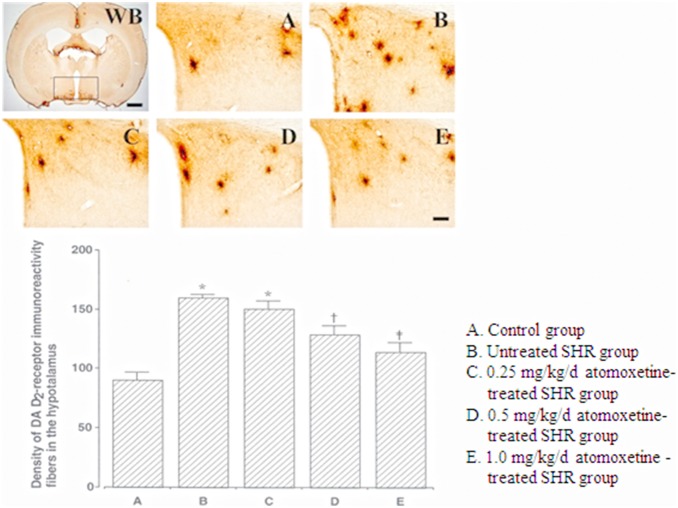
Effect of atomoxetine on DA D_2_ receptor expression in the hypothalamus. Upper: Photomicrographs of DA D_2_ receptor expression in the hypothalamus. WB: Whole brain. The sections were stained for DA D_2_ receptor immunoreactivity (brown). The scale bars represent 50 µm (WB) and 200 µm (A–E). Lower: DA D_2_ receptor expression in each group *p<0.05 compared to the control group. ^†^ *p*<0.05 compared to the untreated ADHD group. ^‡^ *p*<0.05 compared to the untreated ADHD and 0.25 mg/kg/d atomoxetine-treated ADHD groups. The data have been presented as mean ±SEM.

The D_2_ receptor-positive cell density in the hypothalamus was 90.00±0.68 in the control group, 160.20±2.35 in the untreated SHR group, 150.20±1.67 in the 0.25 mg/kg atomoxetine-treated SHR group, 120.80±0.74 in the 0.5 mg/kg atomoxetine-treated SHR group, and 114.40±0.77 in the 1 mg/kg atomoxetine-treated SHR group (Fig. 4). These results showed that the D_2_ receptor- immunoreactive fiber density in the hypothalamus of SHRs was significantly higher than that in the hypothalamus of controls, and atomoxetine treatment significantly and dose-dependently decreased the number of D_2_ receptor-positive cells (*F_(4,40)_* = 65.232, *p*<0.001).

## Discussion

The hyperactive motor activity in the experimental animals improved as the atomoxetine concentration increased: 1 mg/Kg dosage seems to be more accurate than 0.25 mg/Kg or 0.5 mg/Kg. Interestingly, while the improvement in motor activity in the 0.25 mg/Kg and 0.5 mg/Kg groups did not continue after 2 weeks of treatment, the 1 mg/Kg/day group displayed continuous improvement in motor activity even after 2 weeks. This coincides with the clinical findings, demonstrating slower responses to atomoxetine than to cerebral stimulants. In other words, the effects of atomoxetine can be seen after slow titration and optimal dosage. For improving the motor activity in SHRs, these results indicate one of the reasons why pharmacotherapy is less effective than expected, which is inadequate dosage level for treating the symptoms [21].

Current medications for ADHD management mostly aim to control brain levels of dopamine and norepinephrine [2]. The alleviation of ADHD symptoms via atomoxetine treatment probably correlates with changes in NA and DA levels in the PFC, thereby enhancing cognitive function in ADHD patients [22]. In addition, atomoxetine has been demonstrated to increase the expression of tyrosine hydroxylase-positive cells in the PFC and substantia nigra [23]. Atomoxetine treatment has also been reported to suppress DA D_2_ receptor density in the striatum and PFC, while NA stimulation increased signaling, by acting preferentially on the DA D_1_ receptors in the PFC [23]. Although studies focusing on the relationship between the DA D_2_ receptor and ADHD are rare, Bowton et al. [8] emphasized that the DA D_2_ receptor acts as a key modulator of DA stimulation in the dopaminergic system. In this experiment, with respect to DA D_2_ receptor immunohistochemistry, we observed significantly increased DA D_2_ receptor expression in the PFC, striatum, and hypothalamus of the SHRs as compared to the WKY rats. These findings demonstrated that DA D_2_ receptor expression was significantly upregulated in the SHRs than in the control rats. That is, the dopamine synthesis in the PFC, striatum, and hypothalamus of the SHRs was lower than that in the PFC, striatum, and hypothalamus of the control rats. However, the experiment with DAT-KO mice treated with atomoxetine by Del'guidice et al. [16] showed amelioration of cognitive performance without change in hyperactivity.

In summary, the motor activity in SHRs was decreased in accordance with the dosage of atomoxetine. This study suggests that atomoxetine increases DA concentrations in the PFC, striatum, and hypothalamus, resulting in downregulation of the DA D_2_ receptor expression in SHRs.

## References

[1] Brown RT, Freeman WS, Perrin JM, Stein MT, Amler RW, et al (2001) Prevalence and assessment of attention-deficit/hyperactivity disorder in primary care settings. Pediatrics 107(3): E43. 11230624 10.1542/peds.107.3.e43

[2] Dopheide JA, Pliszka SR (2009) Attention-deficit-hyperactivity disorder: an update. Pharmacotherapy 29(6): 656–679. 19476419 10.1592/phco.29.6.656

[3] Genro JP, Kieling C, Rohde LA, Hutz MH (2010) Attention-deficit/hyperactivity disorder and the dopaminergic hypothesis. Expert Rev Neurother 10: 587–601. 20367210 10.1586/ern.10.17

[4] Prediger RD, Pamplona FA, Fernandes D, Takahashi RN (2005) Caffeine improves spatial learning deficits in an animal model of attention deficit hyperactivity disorder (ADHD) the spontaneously hypertensive rat (SHR). Int J Neuropsychopharmacol 8: 583–594. 15877934 10.1017/S1461145705005341

[5] Durston S, Davidson MC, Thomas KM, Worden MS, Tottenham M, et al (2003) Parametric manipulation of conflict and response competition using rapid mixed-trial event-related fMRI. Neuroimage 20: 2135–2141. 14683717 10.1016/j.neuroimage.2003.08.004

[6] Levy F (1991) The dopamine theory of attention deficit hyperactivity disorder (ADHD). Aust NZ J Psychiatry 25: 277–283. 10.3109/00048679109077746 1652243

[7] Missale C, Nash SR, Robinson SW, Jaber M, Caron MG (1998) Dopamine receptors: from structure to function. Physiol Rev 78(1): 189–225. 9457173 10.1152/physrev.1998.78.1.189

[8] Bowton E, Saunders C, Erreger K, Sakrikar D, Matthies HJ, et al (2010) Dysregulation of dopamine transporters via dopamine D_2_ autoreceptors triggers anomalous dopamine efflux associated with attention-deficit hyperactivity disorder. J Neurosci 30: 6048–6057. 20427663 10.1523/JNEUROSCI.5094-09.2010 PMC2881830

[9] Wang M, Pei L, Fletcher PJ, Kapur S, Seeman P, et al (2010) Schizophrenia, amphetamine-induced sensitized state and acute amphetamine exposure all show a common alteration: increased dopamine D2 receptor dimerization. Mol Brain 3: 25. 20813060 10.1186/1756-6606-3-25 PMC2942879

[10] Gründer G (2010) Cariprazine, an orally active D_2_/D_3_ receptor antagonist, for the potential treatment of schizophrenia, bipolar mania and depression. Curr Opin Investig Drugs 11: 823–832. 20571978

[11] Marcil J, Thibault C, Anand-Srivastava MB (1997) Enhanced expression of Gi-protein precedes the development of blood pressure in spontaneously hypertensive rats. J Mol Cell Cardiol 29: 1009–1022. 9152862 10.1006/jmcc.1996.0343

[12] Bymaster FP, Katner JS, Nelson DL, Hemrick-Luecke SK, Threlkeld PG, et al (2002) Atomoxetine increases extracellular levels of norepinephrine and dopamine in prefrontal cortex of rat: a potential mechanism for efficacy in attention deficit/hyperactivity disorder. Neuropsychopharmacology 27: 699–711. 12431845 10.1016/S0893-133X(02)00346-9

[13] Levy F (2008) Pharmacological and therapeutic directions in ADHD: Specificity in the PFC. Behav Brain Funct 28(4): 12. 10.1186/1744-9081-4-12 PMC2289834 18304369

[14] Moreno M, Economidou D, Mar AC, López-Granero C, Caprioli D, et al (2013) Divergent effects of D_2_/_3_ receptor activation in the nucleus accumbens core and shell on impulsivity and locomotor activity in high and low impulsive rats. Psychopharmacology (Berl) 228(1): 19–30. 23407782 10.1007/s00213-013-3010-3 PMC3676742

[15] Fernando AB, Economidou D, Theobald DE, Zou MF, Newman AH, et al (2012) Modulation of high impulsivity and attentional performance in rats by selective direct and indirect dopaminergic and noradrenergic receptor agonists. Psychopharmacology (Berl) 219(2): 341–352. 21761147 10.1007/s00213-011-2408-z PMC3249163

[16] Del'guidice T, Lemasson M, Etiévant A, Manta S, Magno LA, et al (2014) Dissociations between cognitive and motor effects of psychostimulants and atomoxetine in hyperactive DAT-KO mice. Psychopharmacology (Berl) 231(1): 109–122. 23912772 10.1007/s00213-013-3212-8

[17] Davids E, Zhang K, Tarazi FI, Baldessarini RJ (2003) Animal models of attention-deficit hyperactivity disorder. Brain Res Rev 42: 1–21. 12668288 10.1016/s0165-0173(02)00274-6

[18] Wu L, Zhao Q, Zhu X, Peng M, Jia C, et al (2010) A novel function of microRNA let-7d in regulation of galectin-3 expression in attention deficit hyperactivity disorder rat brain. Brain Pathol 20: 1042–1054. 20557304 10.1111/j.1750-3639.2010.00410.x PMC8094722

[19] Ueno KI, Togashi H, Mori K, Matsumoto M, Ohashi S, et al (2002) Behavioral and pharmacological relevance of stroke-prone spontaneously hypertensive rats as an animal model of a developmental disorder. Behav Pharmacol 13: 1–13. 11990715 10.1097/00008877-200202000-00001

[20] Durand M, Berton O, Aguerre S, Edno L, Combourieu I, et al (1999) Effects of repeated fluoxetine on anxiety-related behaviours, central serotonergic systems, and the corticotropic axis axis in SHR and WKY rats. Neuropharmacology 38(6): 893–907. 10465693 10.1016/s0028-3908(99)00009-x

[21] Scahill L, Oesterheld JR, Martin A (2007) General principles, specific drug treatments, and clinical practice. In: Martin A, Volkmar FR, editors. Lewis’s child and adolescent psychiatry: A comprehensive textbook, 4^th^ ed. Philadelphia: Lippincott Williams & Wilkins. 754–756.

[22] Stahl SM (2003) Neurotransmission of cognition, part 2. Selective NRIs are smart drugs: exploiting regionally selective actions on both dopamine and norepinephrine to enhance cognition. J Clin Psychiatry 64: 110–111. 12633117 10.4088/jcp.v64n0201

[23] Levy F (2009) Dopamine vs noradrenaline: inverted-U effects and ADHD theories. Aust N Z J Psychiatry 43: 101–108. 19153917 10.1080/00048670802607238

